# Investigating the impacts of recycled water on long-lived conifers

**DOI:** 10.1093/aobpla/plv035

**Published:** 2015-04-15

**Authors:** Lloyd L. Nackley, Corey Barnes, Lorence R. Oki

**Affiliations:** Department of Plant Sciences, University of California, Davis, CA 95616, USA

**Keywords:** California, drought, Mediterranean climate, reclaimed water, urban forestry, urban horticulture

## Abstract

Recycled wastewater is a popular alternative water resource. Recycled water typically has higher salinity than potable water and therefore may not be an appropriate water source for landscapes planted with salt-intolerant plant species. Coast redwoods (*Sequoia sempervirens*) are an important agricultural, horticultural and ecological species assumed to be salt intolerant. However, no studies have analysed how salinity impacts coast redwood growth. To determine salt-related growth limitations, as well as susceptibility to particular salt ions, we divided 102 *S. sempervirens* ‘Aptos Blue’ saplings evenly into 17 salinity treatments: a control and four different salts (sodium chloride, calcium chloride, sodium chloride combined with calcium chloride, and sodium sulfate). Each salt type was applied at four different concentrations: 1.0, 3.0, 4.5 and 6.0 dS m^−1^. Trees were measured for relative growth, and leaves were analysed for ion accumulation. Results showed that the relative stem diameter growth was inversely proportional to the increase in salinity (electrical conductivity), with *R*^2^ values ranging from 0.72 to 0.82 for different salts. Analysis of variance tests indicated that no particular salt ion significantly affected growth differently than the others (*P* > 0.1). Pairwise comparisons of the means revealed that moderately saline soils (4–8 dS m^−1^) would decrease the relative height growth by 30–40 %. Leaf tissue analysis showed that all treatment groups accumulated salt ions. This finding suggests reduced growth and leaf burn even at the lowest ion concentrations if salts are not periodically leached from the soil. Regardless of the specific ions in the irrigation water, the results suggest that growth and appearance of coast redwoods will be negatively impacted when recycled water electrical conductivity exceeds >1.0 dS m^−1^. This information will prove valuable to many metropolitan areas faced with conserving water while at the same time maintaining healthy verdant landscapes that include coast redwoods and other long-lived conifers.

## Abstract (precis)

This research has direct implications for public and private institutions seeking to conserve water by irrigating landscapes with recycled (a.k.a. reclaimed) water. Although typical salt contents in recycled water are low (< 2.0 dS m^−1^), levels may still be harmful to salt-sensitive plants. We discovered that salt accumulation in soils would negatively impact coast redwoods when recycled water salinity exceeds>1.0 dS m^−1^. This is the first paper reporting the impacts of salinity on the growth of the coast redwood. The results suggest that irrigation management of long-lived conifers will be essential to protect these important trees.

## Introduction

Water used to irrigate important verdant, social landscapes (e.g. arboreta, public parks and golf courses) faces competition with other uses of fresh water including increasing agricultural and urban demands (Hamilton *et al*. 2005). Recycled wastewater has been highlighted as one of the most affordable alternative resources for agricultural, industrial and urban non-potable purposes in arid and semi-arid regions like California, where current fresh water reserves are at a critical limit (Lazarova *et al*. 2001). In California natural prolonged periods of summer drought have been exacerbated in recent years by low winter rainfall. California's 2014 Water Year, which ended 30 September 2014 was the third driest in 199 years of record; and was the warmest year on record (USGS 2015). In addition, California's population is estimated to increase by 15.4 million residents (a 39 % increase) over the next 50 years (Palmer and Schooling 2013). Both the rise in population and the uneven distribution of these new inhabitants will cause an increase in water demand (Hanak and Davis 2006). To mitigate the effects of increased competition for limited potable water, horticulturalists and municipalities in California and in arid and semi-arid climates around the world are developing sources of recycled wastewater (Hamilton *et al*. 2005; Miller 2006; Toze 2006).

Types of wastewaters used for recycling include treated and untreated sewage effluent, storm water runoff, domestic greywater and industrial wastewater (Toze 2006). Although recycled water meets many social and environmental objectives by reducing competition for fresh water, there are some drawbacks that make it less suitable than potable water for horticultural applications. Primarily, recycled water often has a greater salt concentration than potable water. Although the salinity of recycled water is not usually high enough to make it unsuitable for irrigation (Vartanian 2008), it can contain 10 times more salt (e.g. 1.0–2.0 dS m^−1^) than potable water (∼0.1 dS m^−1^). Thus, recycled water can be harmful to salt-intolerant plants (Maas 1986).

Salinity in low concentrations (<2.0 dS m^−1^) has been shown to have adverse effects on growth and physiology of many plants (Kozlowski 1997; Chaves *et al*. 2009). Salinity impacts plant growth by decreasing the osmotic potential of the soil and imposing physiological drought, or through toxic effects from high concentrations of particular ions, such as sodium or chloride that can injure the plant (Chaves *et al*. 2009). Although there is an extensive literature on the negative effect of salt on plant growth for many agricultural crops (Sohan *et al*. 1999; Sultana *et al*. 1999; Katerji *et al*. 2003; Zheng *et al*. 2008), there is a limited amount of information quantifying growth responses to salt for important horticultural species. In particular, there is only one report published about the salt tolerance of the coast redwood tree (*Sequoia sempervirens*) (Wu and Guo 2006), which is surprising given this species’ important ecological and horticultural value.

**Figure 1.**
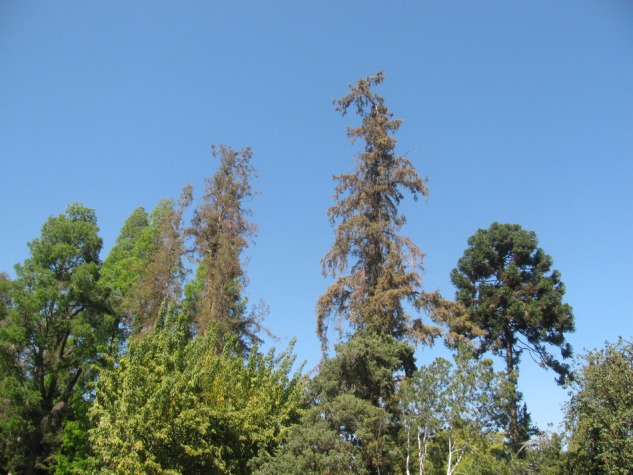
A photograph of coast redwoods (*Sequoia sempervirens*) that are growing in a public park in the San Francisco Bay Area, CA. The coast redwoods are the tall, slender conifers, exhibiting characteristic leaf senescence (browning) and stem dieback. The cause of stress of these trees was identified as water stress. Water stress can be caused by lack of soil moisture, or physiological drought from increased soil salinity.

The coast redwood is emblematic of western US conifers known for its towering height (>100 m) and longevity (>1500 years). This charismatic tree species’ native range extends along the fog-belt of the Pacific coast from southern Oregon to central California. The coast redwood is an important timber species, prized in building for its burnt-sienna coloured wood that is naturally decay resistant. Coast redwoods are also used extensively in Pacific horticulture (CA, OR, WA), in public parks, golf courses, highways and private landscapes; and are popular horticultural specimens used throughout the USA and in temperate climates around the world. Although the coast redwood is indigenous within a Mediterranean climate, which is typified by long periods of summer drought, coast redwoods thrive in areas with significant summertime moisture, typically derived from abundant marine fog. Moisture input from fog drip in the summer can constitute 30 % or more of the total water input each year (Dawson 1998). The coast redwood is characterized as having low to moderate drought tolerance (Sunset Books 2000) and requires supplemental irrigation where fog or summer precipitation events are lacking. Without natural precipitation (rain or fog) or supplemental irrigation, dry summer conditions may inhibit the performance of mature individuals of coast redwood in urban settings where signs of water stress often include leaf senescence and stem die back (Litvak *et al*. 2011) (Fig. 1).

The work presented herein was initiated to fill a knowledge gap by determining the level of tolerance of coast redwood to sodium and chloride. The research was designed in response to reports from water districts in the San Francisco Bay Area, which claimed that coast redwoods within public parks had shown signs of decline or death after irrigation with recycled water. To determine the effects of sodium and chloride ions on the growth and health of redwoods, *Sequoia sempervirens* ‘Aptos Blue’ specimens were placed in a greenhouse and irrigated daily with one of 17 treatments represented by a non-saline nutrient solution that was used as the control treatment plus four different salt solutions at four different concentrations. We hypothesized that redwoods would be classifiable as a ‘salt-sensitive’ species, demonstrated by declines in growth at soil salinity concentrations <3.0 dS m^−1^. Further, we hypothesized that different salt solutions would be more toxic than others, represented by statistically different growth responses.

## Methods

### Experimental design

The experiment was conducted in a glasshouse at the UC Davis Environmental Horticulture Complex (Davis, CA, USA). Greenhouse daytime low and high temperatures were maintained between 21 and 24 °C, and night-time low and high temperatures were maintained between 13 and 17 °C. No artificial lighting was supplied to the plants. The glasshouse was divided into two blocks to control for natural gradients of sunlight, temperature and humidity. Pots were placed 1 m apart throughout the two blocks. One hundred and two *Sequoia sempervirens* ‘Aptos Blue’ saplings in 8 L pots (21 cm tall, with a 21 cm diameter tapering to 18.5 cm) were obtained from Generation Growers, Modesto, CA, USA. Potting media contained a mix of humus and sand in a 4 : 1 volumetric ratio, 6.0 kg m^−3^ dolomite, 0.6 kg m^−3^ calcium nitrate, 1.2 kg m^−3^ ferrous sulfate heptahydrate, 3.0 kg m^−3^ nitroform, 2.4 kg m^−3^ double super phosphate and 1.2 kg m^−3^ oyster shell lime.

The salinity treatments consisted of a control, as well as four different salts: sodium chloride (NaCl), calcium chloride (CaCl_2_), sodium chloride and calcium chloride (NaCl + CaCl_2_) and sodium sulfate (Na_2_SO_4_). Each salt was applied at four different concentrations represented by electrical conductivity (EC) of 1.0, 3.0, 4.5 and 6.0 dS m^−1^. NaCl was selected because it is the most common salt in recycled water. Na_2_SO_4_ was used to isolate Na symptoms, whereas CaCl_2_ served to isolate Cl symptoms. The combination of NaCl and CaCl_2_ provided a treatment simulating environmental conditions, where combinations of monovalent and multivalent cations would be present in the irrigation water and/or soil. Each salt type was added to a one-quarter strength Hoagland's fertilizer ‘Solution 2’ which had an EC of 0.5 dS m^−1^ (Epstein and Bloom 2005). The control treatment received only the modified Hoagland’s, without additional salt. Six trees were replicated in each of 17 treatments. Treatments were initialized on 15 October 2005.

Dosatron^®^ DI-16 injectors (Dosatron USA, Clearwater, FL, USA) were used to mix the salinity treatments into the irrigation water. Three Netafim^®^ Woodpecker pressure-compensating emitters (Netafim Irrigation, Fresno, CA, USA, rated 4 L h^−1^) at each pot produced an average total flow rate of 12.8 L h^−1^ (SE = 0.08, *n* = 9). Multiple emitters at each pot allowed for uniform saturation of the container medium. Daily irrigations were scheduled with a Hunter^®^ ICC irrigation timer (Hunter Industries Inc., San Marcos, CA, USA). A leaching fraction of 0.4–0.5 was applied to all treatments independently. The leaching fraction is defined as the ratio of the quantity of water draining past the root zone to that infiltrated into the soil's surface. This fraction was used to isolate symptoms related to the salt treatments by eliminating stress due to both insufficient water and increasing container EC due to evapotranspiration. Further, this leaching fraction was designed to provide sufficient irrigation treatment volume to allow for uniform saturation of the container medium. Irrigation treatment salinity concentrations were evaluated weekly by collecting solute from the emitter tube at each tree during the day's irrigation cycle. After the irrigation cycle, a portable meter was used to test the EC and pH of each sample leachate (Table 1).

**Table 1.** Mean (±SE) cumulative treatment and leachate EC values from the testing period 12 July 2005 to 1 September 2007. A leaching fraction of 0.4–0.5 was applied to all treatments independently. Irrigation treatment salinity concentrations were evaluated weekly by collecting solute from an emitter tube at each tree prior to the day's irrigation cycle.

Treatment	Cumulative mean treatment EC (dS m^−1^) ± 1 SE	Cumulative mean leachate EC (dS m^−1^) ± 1 SE
Control 0.5 dS m^−1^	0.57 ± 0.01	0.66 ± 0.01
NaCl 1.0 dS m^−1^	1.05 ± 0.01	1.67 ± 0.05
NaCl 3.0 dS m^−1^	3.12 ± 0.03	4.52 ± 0.11
NaCl 4.5 dS m^−1^	4.32 ± 0.05	5.71 ± 0.11
NaCl 6.0 dS m^−1^	5.72 ± 0.08	7.08 ± 0.12
CaCl_2_ 1.0 dS m^−1^	1.06 ± 0.01	1.54 ± 0.02
CaCl_2_ 3.0 dS m^−1^	2.95 ± 0.02	5.08 ± 0.13
CaCl_2_ 4.5 dS m^−1^	4.52 ± 0.04	7.10 ± 0.16
CaCl_2_ 6.0 dS m^−1^	6.12 ± 0.04	8.83 ± 0.17
NaCl + CaCl_2_ 1.0 dS m^−1^	1.09 ± 0.01	1.61 ± 0.03
NaCl + CaCl_2_ 3.0 dS m^−1^	2.94 ± 0.03	4.60 ± 0.11
NaCl + CaCl_2_ 4.5 dS m^−1^	4.59 ± 0.03	6.83 ± 0.16
NaCl + CaCl_2_ 6.0 dS m^−1^	6.10 ± 0.04	8.40 ± 0.15
Na_2_SO_4_ 1.0 dS m^−1^	1.09 ± 0.01	1.73 ± 0.05
Na_2_SO_4_ 3.0 dS m^−1^	3.10 ± 0.04	4.68 ± 0.11
Na_2_SO_4_ 4.5 dS m^−1^	4.71 ± 0.01	6.08 ± 0.09
Na_2_SO_4_ 6.0 dS m^−1^	6.10 ± 0.02	7.37 ± 0.11

### Data collection

Stem diameter and stem length (i.e. tree height) were measured every second week starting on 25 September 2005 and ending 3 January 2007. A set of digital calipers (Fisher Scientific, Pittsburgh, PA, USA) was placed around the trunk at a height of 3 cm above the potting medium in a constant orientation for each tree. The trunk was marked to indicate the points of contact for the calipers and the diameter was measured across these points each time. Tree height was evaluated every third week starting 15 September 2005 and ending 8 January 2007. Height was measured with a tape from an indicated point on the pot rim to the apex of the central leader of the tree.

The concentration of salt ions accumulated in the leaves was determined from analyses of leaves sampled from the previous flush of growth. These leaves were identified as originating from lignifying stem segments occurring directly behind the youngest, light green leaves on solid green stems. Consistency of tissue maturity has been shown to be an important characteristic for obtaining comparable results (Mills and Jones 1996). Leaf tissue-sampling events occurred on 17 October 2005, 9 January 2006, 18 May 2006, 22 September 2006 and 15 January 2007. The experiment was terminated shortly after the fifth sampling. Both proximal (P) and distal (D) leaf blade sections were collected on each date. The distal portions of leaves were removed first; the halfway cut point was determined visually. Then the basal sections of the cut leaves were removed by cutting them as closely to the stem as possible. A minimum of 1.5 g dry weight (3.8 g fresh weight, 39 % dry: fresh weight ratio) was collected for each sample. The dried samples were analysed for % Ca^+^, % Cl^−^ and % Na^+^ by using the ‘Nitric/Perchloric Wet Ashing Open Vessel’ (P – 3.10) technique, and Cl was analysed using the ‘2 % Acetic Acid Extraction’ (P – 4.20) technique by Dellavalle^®^ Laboratory, Inc. (Fresno, CA, USA). Ion accumulation rates were evaluated for the different concentrations within each salinity treatment type (e.g. 1.0 dS m^−1^ NaCl vs. 6.0 dS m^−1^ NaCl), as well as within treatment concentration level between the various salinity treatment types (e.g. 1.0 dS m^−1^ NaCl vs. 1.0 dS m^−1^ CaCl_2_).

### Statistical analysis

**Figure 2.**
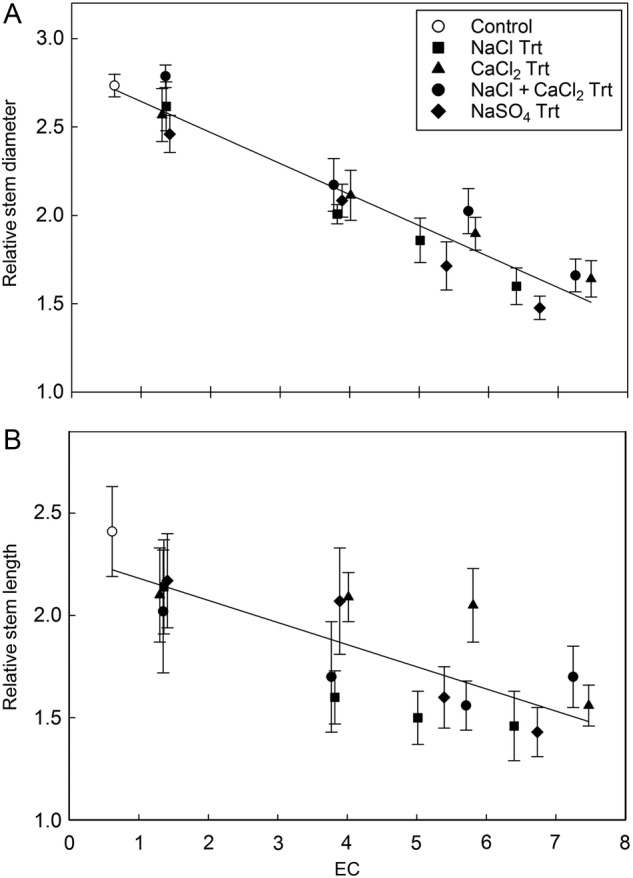
(A) Irrespective of salt ion the relative diameter growth was inversely proportional to the increase in salinity (*R*^2^ = 0.75), which was measured as the mean of the irrigation and leachate EC. The *R*^2^ relationship between EC and relative diameter growth values for the control and four concentrations within each salinity type ranged from 0.72 for the CaCl_2_ treatments to 0.82 for the Na_2_SO_4_ treatments. Analysis of variance tests indicated that the specific salt (or ion combination) did not have a significantly greater effect on the diameter growth of the stem (*P* > 0.1). (B) The specific ion (or ion combination) did not have a significantly greater effect on the vertical growth of the stem (*P* > 0.1). The relative stem growth was poorly explained as a response to increasing salinity (EC) (*R*^2^ = 0.27). Yet, there were significant differences in height (*P* < 0.001) between the different concentration levels within the salt treatment. Pairwise multiple comparisons of the means suggest that the final relative height in the control group and the lowest ion concentration (1.0 dS m^−1^) were significantly greater (*P* < 0.05) when compared with the two higher ion concentrations (4.5 and 6.0 dS m^−1^).

**Figure 3.**
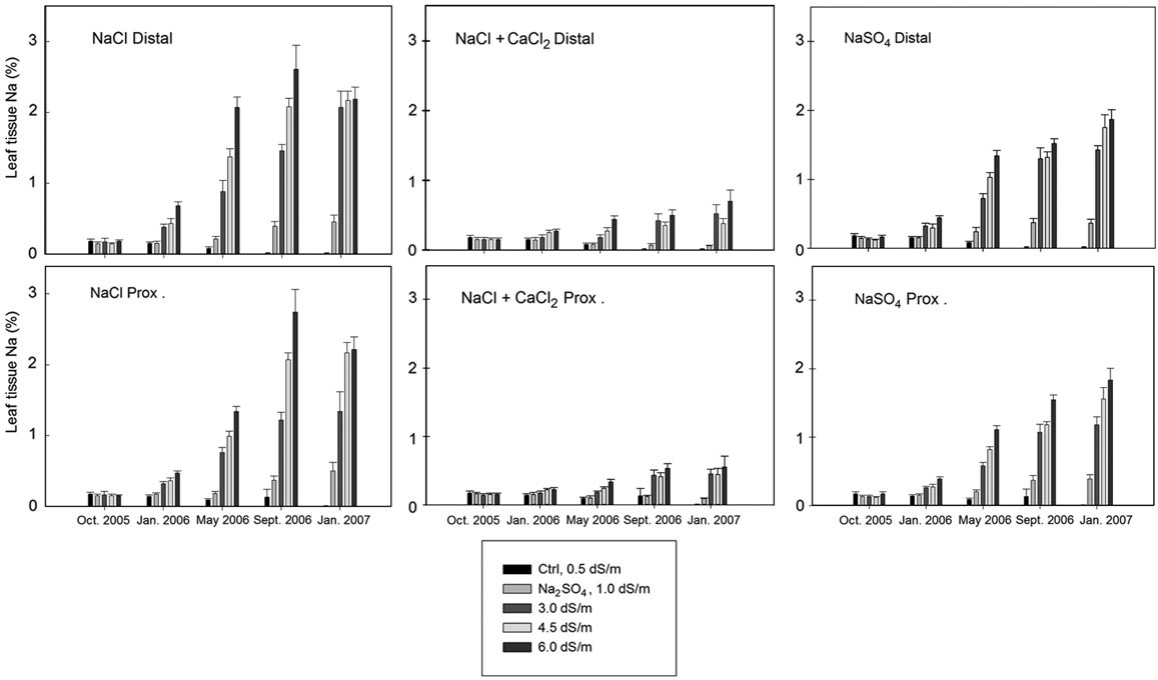
Na^+^ ion concentration in the distal and proximal ends of the leaf tips from all treatment groups (excluding the CaCl_2_ treatments). At every harvest the greatest % Na^+^ in leaf tissues (distal and proximal) were detected in the 6.0 dS m^−1^ group in NaCl treatment. Over time, the % Na tissue concentration increased in all salinity concentrations across all ion treatments. The per cent concentration did not differ greatly between distal and proximal ends of the leaf for any treatment combination. The distal and proximal leaf tissues harvested from plants treated with NaCl had 2–3 times greater Na^+^ when compared with the NaCl + CaCl_2_ group, and 25 % greater than the NaSO_4_ group.

**Figure 4.**
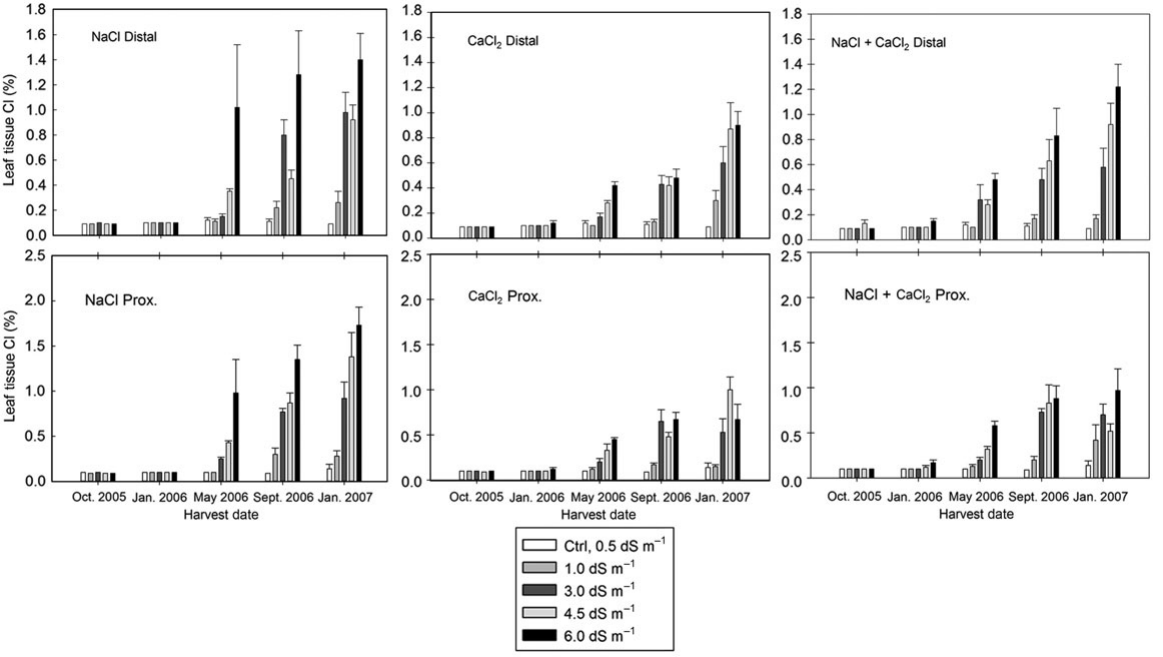
The rate of Cl^−^ increase was relative to the salinity concentration rate in the irrigation solution, with the greatest tissue concentrations in the plants in treatment groups with the greatest ions. The % Cl^−^ concentration did not differ greatly between distal and proximal ends of the leaf for any treatment combination. The Cl^−^ concentration did not initially increase (October 2005–January 2006). However, subsequent harvests exhibited increasing concentration of Cl^−^ ions in the leaf tissues.

**Figure 5.**
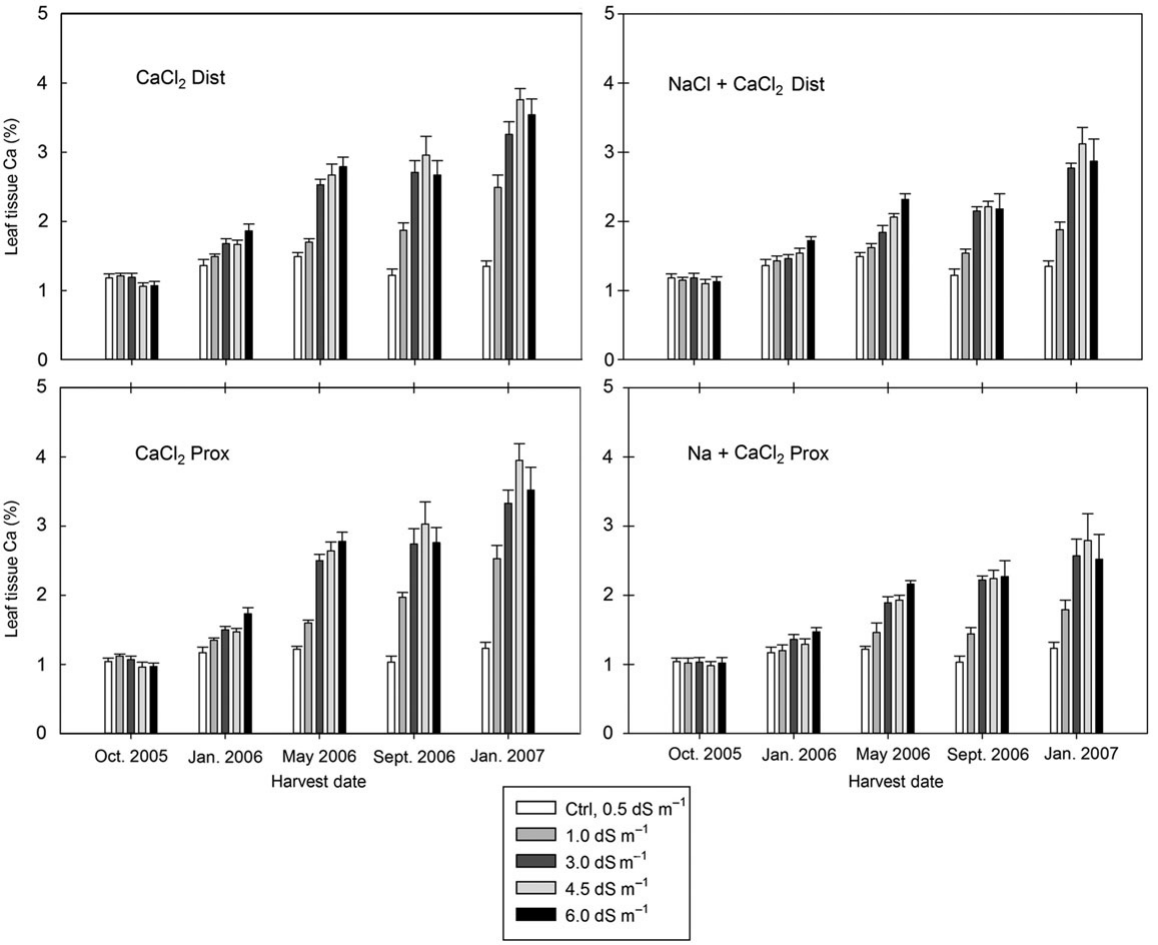
Calcium ion concentration in the leaf tissues followed the same pattern as Na^+^ and Cl^−^, with similar concentrations being found in distal and proximal segments and the greatest concentrations being relative to the treatment concentrations, which increased with exposure over time. The final concentrations of Ca^+^ ions found in the leaf tissues in January 2007 were >2 % for all Ca^+^ containing salt treatments. Conversely, no treatment had >2 % Cl^−^, and only the 6.0 NaCl treatments had >2 % Na.

The experimental design represented a randomized complete block (RCB) with every treatment appearing in either block. Blocking of experimental groups in the greenhouse was used to control for variances that may have originated from the environmental conditions. The treatment and block effects were treated as random variables. Analysis of variance (ANOVA) tests were used to evaluate if differences in stem (height) growth and stem diameter growth could be confidently attributed (*P* < 0.05) to blocking, salinity type and salinity concentration. For each salt type, a linear regression was used to determine the influence increasing salinity concentrations (EC) had on height and diameter growth responses (Fig. 2). Tukey's HSD were used as a post-hoc pairwise comparison of means within treatments when statistical differences were attributable to the treatment variables (salt or concentration). Relative comparisons of the mean ion concentrations, collected in proximal and distal tissues, were used to understand the differences attributable to salinity type and salinity concentration (Figs 3–5).

## Results

### Growth

The stem length (height) and diameter measurements were highly dependent on the natural variability of initial starting size rather than the treatment effects. To elucidate the treatment effects we investigated the relative growth for each tree, rather than the absolute growth. Relative growth was calculated by dividing the recorded height or diameter measurements by the original (9 September 2005) height or diameter measurements. Analysis of variance results suggest no significant differences (*P* > 0.1) attributable to the experimental blocks. The magnitude of salinity effects, measured as mean EC from the cumulative irrigation EC and the cumulative leachate EC, was similar for all four salt treatments. Trees grown at 6.0 dS m^−1^ increased ∼1.5 times the original diameter. Trees grown at 1.0 dS m^−1^ increased ∼2.5 times the original diameter (Fig. 2A), which was similar to the growth by the control group. The proportion of the variation in stem diameter growth responses explained by increasing salinity (EC) ranged from *R*^2^ = 0.72 for the CaCl_2_ treatments, *R*^2^ = 0.74 for NaCl + CaCl_2_, *R*^2^ = 0.77 NaCl, to *R*^2^ = 0.82 for the Na_2_SO_4_ treatment. Analysis of variance tests indicated that the specific salt (or ion combination) did not have a significantly greater effect on the diameter growth of the stem (*P* > 0.1). An ANOVA testing the response of height growth to the salt treatments suggested significant differences (*P* < 0.001) between the different concentration levels within the salt treatments.

Means of all the treatments showed an inverse relationship between stem length growth and increasing EC (Fig. 2B). Yet, the mean relative stem length and the corresponding standard errors overlapped greatly. Intermediate salinity concentrations caused the most variation within and between treatments in relative tree height (Fig. 2B). Relationships between EC and relative height was poorly described by linear modelling with *R*^2^ values for each salinity type ranging from 0.18 for the NaCl + CaCl_2_ treatments, *R*^2^ = 0.2 CaCl_2_, *R*^2^ = 0.34 NaSO_4_, to 0.38 for the NaCl treatment. A Tukey HSD pairwise comparison revealed that the final relative height (15 January 2007) of the control group (2.4 ± 0.2) was not significantly greater (*P* > 0.1) than the final relative height of any of the lowest salinity concentration (1.0 dS m^−1^) (NaCl: 2.1 ± 0.2; CaCl_2_: 2.1 ± 0.2, NaCl + CaCl_2_: 2.0 ± 0.3; Na_2_SO_4_: 2.2 ± 0.2). Yet Tukey HSD pairwise comparisons suggest that the final relative height in the control group and the lowest ion concentration (1.0 dS m^−1^) were significantly greater (*P* < 0.05) when compared with the two higher ion concentrations (4.0 and 6.0 dS m^−1^). The significant differences between the high and low salinity groups suggest that if recycled water generates moderately saline soils (4–8 dS m^−1^) that the relative height growth of coast redwoods may decrease by 30–40 %.

### Tissue analysis

At every harvest, the greatest % Na^+^ in leaf tissues (D or P) was detected in the 6.0 dS m^−1^ group in NaCl treatment (Fig. 3). The per cent concentration did not differ greatly between D or P leaf sections for any treatment combination and hereafter will not be differentiated. Over time, the % Na^+^ tissue concentration increased in all salinity concentrations across all ion treatments (excluding CaCl_2_, which did not contain Na^+^ ions). The leaf tissues harvested from plants treated with NaCl had 2–3 times greater Na^+^ when compared with the NaCl + CaCl_2_ group, and 25 % greater than the NaSO_4_ group.

Similar to the Na^+^ tissue analyses, the rate of Cl^−^ increase was relative to the salinity concentration in the irrigation solution with the greatest tissue concentrations occurring in the plants in treatment groups with the greatest ions concentration (Fig. 4). Like Na^+^, the % Cl^−^ concentration did not differ greatly between D and P ends of the leaf for any treatment combination. The leaf Cl^−^ concentration did not initially increase (October 2005–January 2006). However, subsequent harvests exhibited increasing concentrations of Cl^−^ ions in the leaf tissues for all treatments containing Cl^−^ ions.

Calcium ion concentration in the leaf tissues followed the same pattern as Na^+^ and Cl^−^, with similar concentrations being found in D and P segments and the greatest concentrations being relative to the treatment ion concentrations, which increased with exposure over time (Fig. 5). A notable difference between the % Na^+^ (Fig. 3), % Cl^−^ (Fig. 4) and % Ca^+^ (Fig. 5) analyses is that the % Ca^+^ in the control group was nearly an order of magnitude greater than either of the other two ions (1 % compared with 0.1 %). Corresponding to this relatively increased baseline, the final concentrations of Ca^+^ ions found in the leaf tissues at January 2007 were >2 % for all Ca^+^ containing salt treatments. Conversely, no treatment had >2 % Cl^−^, and only the 6.0 NaCl treatments had >2 % Na.

## Discussion

### Growth responses

The impetus of the study was to quantify the toxicity of Na^+^ and Cl^−^ to coast redwood, since horticultural problems had been observed in coast redwoods irrigated with recycled water salinity in the Santa Clara, CA, USA area, and NaCl is the primary salt in recycled water in the Santa Clara, CA, USA, area. The salinity of recycled water may be the single most important parameter in determining its suitability for irrigation (EPA 2012). Salinity limits vegetative and reproductive growth of plants by inducing severe physiological dysfunctions and causing widespread direct and indirect harmful effects, even at low salt concentrations (Kozlowski 1997). Although the salts present in recycled water can vary greatly depending on the water source, the more common elemental ions include B^3+^, Ca^2+^, Cl^−^, Na^+^ and $\text{S}{{\text{O}}_{4}}^{-}$ (Martinez and Clark 2012).

The treatments were designed to isolate particular ion effects: NaCl, the salt of interest, CaCl_2_ to isolate the chloride effect, Na_2_SO_4_ to isolate the sodium effect, and the combination of NaCl + CaCl_2_ to represent a mixture of mono- and divalent cations that would be closer to a ‘real’ exposure. The similar growth patterns and lack of statistical differences (*P* > 0.1) between salt ions at each respective concentration level (Fig. 2) suggest that none of the ions or ion combinations were more toxic than the others for coast redwood growth. Growth was significantly decreased when salinity in irrigation water was >3 dS m^−1^ when compared with water with 1.0 dS m^−1^ levels of salt (*P* < 0.05) (Fig. 2). The diameter growth responses to increasing salinity were fairly uniform with nearly three-quarters or more of the variation (*R*^2^ = 0.72–0.82) being explained by increasing salinity. The height (stem length) responses were also negatively correlated with increasing salinity. Yet scarcely a third, at best, of the variation was attributable to increasing salinity (*R*^2^ = 0.18–0.38). At intermediate salinity concentrations (EC 3.5 and 5.5) it appears (Fig. 2B) that CaCl_2_ was less inhibiting on stem growth than other salts (e.g. NaCl). Yet differences in salt type were not found to be statistically significant determinant of stem growth (*P* > 0.2). The coast redwood is a hexaploid—each of its cells containing six sets of chromosomes, with 66 chromosomes. Natural genotypic variation is incredibly complex in this species and long-lived tree species in general. Additional analyses with greater sample sizes over longer duration could potentially improve the clarity of response between salt type and stem elongation. Nonetheless, the lack of statistical differences in growth reduction between specific salt ions agrees with previous research that did not find specific ion effects on redwood leaf responses to salt spray (Wu and Guo 2006). Confirming that overall salinity reduces growth, more so than specific ion toxicity, is an important finding because it suggests that a variety of sources of recycled water may be suitable for irrigation water in areas where coast redwoods are growing. However, these effects may change over time depending on the soil and climate where the trees are growing.

**Figure 6.**
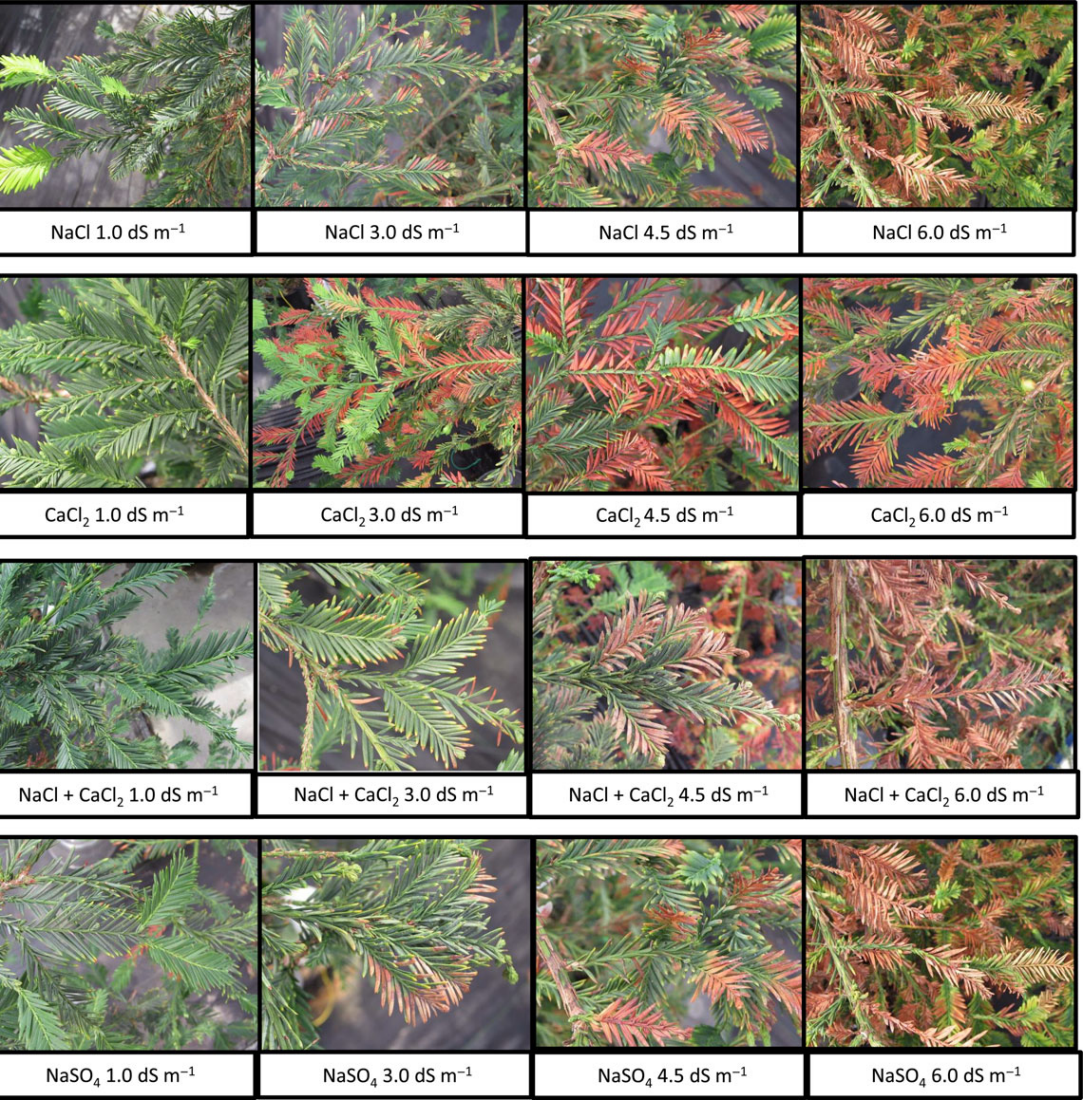
Photographs of the leaves of individual trees taken on 22 September, 2006, ∼1 year after the salinity treatments had been initiated. The visual evidence presented in these photos is characteristic of the leaf burn that was typical for all plants within each particular salt/concentration group. The photos display how little the leaves were affected at 1.0 dS m^−1^, with progressively more burn at higher concentrations.

Salt may become progressively concentrated in the root zone because the plant roots absorb water but very little salt (Kozlowski 1997). In semi-arid and arid environments, saline soils can be problematic due to the lack of adequate rainfall to leach ions from the surface soil layers. The concentration of salt ions can have different effects in different soils. For instance, Na cations are known to interact with anionic clay soils resulting in swelling dispersion of the clay particles, which can reduce soil permeability (Halliwell *et al*. 2001). The buildup of salts in any type of soil will reduce the hydraulic conductivity, which can impact on the ability of water to infiltrate into the soil profile, and thus reduce the water availability to irrigated plants (Toze 2006). Plants will accumulate non-toxic osmolytes in the leaves to counter the change in soil osomotic potential (Greenway and Munns 1980; Munns and Tester 2008). In plants that are unable to exclude the salt, of salt ions, overabundant salt ions will also accumulate in leaf tissue vacuoles (Greenway and Munns 1980; Chaves *et al*. 2009). While this concentration of salt ions in the vacuoles can provide temporary osmotic balance and allow for water uptake, it can also become toxic over time as the ions begin to degrade the chloroplasts (Chaves *et al*. 2009). The statistical differences in growth across the given concentrations (Fig. 2) are even more clearly represented by the visual effects of leaf burn and necrosis (Fig. 6). The continually increasing salt ion concentrations in the leaf tissues (Figs 3–5) suggests that there is the possibility for reduced growth and leaf burn even at the lowest ion concentrations. These results demonstrate that salts must be periodically leached from the soil to prevent the accumulation of greater ionic concentrations in the leaves over time. Salt accumulation in the soil may not be problematic in areas where winter rainfall is sufficient to leach the salt ions. However, this will depend in part on the soil chemistry and structural properties including infiltration and drainage rates.

There has only been one previous study published regarding salt tolerance of coast redwoods (Wu and Guo 2006). Wu and Guo (2006) reported that coast redwood variety Aptos Blue should be placed in the salt sensitive category, because leaves were significantly chlorotic when foliar sprays were applied at a concentration of >2.0 dS m^−1^. The experimental design of their study differed considerably from the work presented herein. Wu and Guo's (2006) salt tolerance categorization was based on the quantity of leaf yellowing after the application of a foliar spray containing salts. Yellowing is an important consideration for horticultural aesthetics, however, because the tolerable level of leaf chlorosis will depend on the management objectives, it is a more subjective measurement compared with the growth responses reported herein. Another important difference is that Wu and Guo's (2006) study focussed on foliar contact, which is important in situations where small trees may be sprayed by overhead irrigation. Yet salt spray on leaves may not be relevant in landscape situations where the spray from an overhead sprinkler is well below the crown of a mature coast redwood. We believe that our results, which analysed impacts of soil salinity, compliment the previous research on foliar responses to salinity. This is especially so considering that if recycled water is being utilized as a means of water conservation, in which case it should be used in conjunction with efficient irrigation methods (e.g. drip or micro spray emitters) (Pereira *et al*. 2002). Foliar spray on mature trees would be unlikely from these more efficient forms of irrigation.

### Management considerations for recycled water in urban landscapes and horticulture

Sodium and other forms of salt are difficult to remove and may be persistent in recycled water (Toze 2006). Reverse osmosis, the most commonly used desalination technology (Fritzmann *et al*. 2007), is typically reserved for the production of high-quality recycled water (i.e. potable water) and would not be practical or economical for landscape irrigation (Toze 2006). Considering that long-term accumulation is a concern for coast redwoods, if salts were present in recycled water, irrigation management would have to rotate between recycled water and fresh water to leach salt ions, utilize recycled water blended with higher quality water to reduce salinity or depend on natural precipitation events. In a container nursery setting—where the soil media is well drained and leached regularly—recycled water with low salinity (<2.0 dS m^−1^) could also be applied safely to irrigate coast redwoods as long as the containers were leached periodically with non-saline water to inhibit accumulation overtime.

The responses demonstrated in our study by the coast redwood, including decreased growth at moderate salinity levels and mortality at high salinity, were similar to responses reported for other conifers (Croser *et al*. 2001). Similar to our finding of increased salt accumulation over time (Figs 3–5), needle burn in ponderosa pine (*Pinus ponderosa* L.) irrigated with recycled wastewater was largely correlated with needle Na^+^ concentration (Qian *et al*. 2005). However, it has been reported that loblolly pine (*Pinus taeda* L.) irrigated with untreated laundry wastewater had improved shoot growth, which the authors attributed to the CaCl_2_ found in the wastewater (Warren *et al*. 2004). Considering our results, these findings may not translate to all conifers. Further, the presence of CaCl_2_ was found to decrease nutrient uptake in Norway spruce (*Picea abies* (L.) Karst.) (Bogemans *et al*. 1989). In general, there are very few studies of the impacts of salinity on conifers, which presents a challenge for mangers using recycled water and an opportunity for future studies. We conclude that *Sequoia sempervirens* ‘Aptos Blue’ was sensitive to salinity regardless of salt composition, particularly as the EC of the irrigation water exceeded 1.0 dS m^−1^ (Figs 2 and 6).

## Conclusions

Fresh water is an essential resource—integral to all ecological and societal activities (Gleick 2012). Growing global human populations must manage finite fresh water resources to meet basic human needs while also ensuring that the extraction of water from natural sources (e.g. rivers, lakes, aquifers etc.) does not deleteriously affect other important ecosystem services derived from freshwater ecosystems (Millennium Ecosystem Assessment 2005).Water reuse for non-potable (i.e. irrigation, industrial) or indirect potable (e.g. discharge into drinking water reservoirs or supply) purposes has been considered across the USA, but particularly in drier or drought-ridden communities such as Arizona, California, Colorado and Texas; or communities experiencing substantial population and economic growth that place a strain on water supplies (e.g. Georgia and Florida) (Hartley 2006).

Unlike monoculture agriculture that manages a single species most landscape plantings include a diverse assemblage of species. Therefore, salt concentrations in recycled water must be acceptable for the most sensitive landscape plant species. The growth responses of the coast redwood to drip irrigation with a variety of salt ions across a range of salinity concentrations had never been reported until this work. This information will prove valuable to the many metropolitan areas faced with conserving water while at the same time maintaining healthy, verdant landscapes that include coast redwoods and other long-lived conifers. For instance, recycled water is a necessity in many metropolitan communities in California, including the San Francisco suburb Redwood City. Redwood City delivers ∼2500 ML year^−1^ of recycled water for municipal irrigation, which is a portion of the 40 150 ML year^−1^ of recycled water provided by the South Bayside System Authority for municipal uses (primarily urban landscapes) (Ingram *et al*. 2006). The EC of this recycled water typically ranges from 1.0 to 1.5 dS m^−1^ (Santa Clara Valley Water District 2007). As the name Redwood City suggests, the coast redwood is an important component of the urban forest, and is the ‘City Tree’ of nearby Palo Alto, CA, USA, a city named for a charismatic *Sequoia sempervirens* ‘El Palo Alto’ (Spanish for tall stick). The results from this study show that like other long-lived conifers, coast redwoods may be able to tolerate the low levels of salt that are typically found in recycled water. In landscapes where interaction between clay soils and saline recycled water may exacerbate salt stress, more drought tolerant/salt tolerant conifers such as pines (e.g. *Pinus palustris* Mill., *P. pinea* L., *P. pinaster* Aiton, and *P. radiata* D. Don) (Antonellini and Mollema 2010) could be suitable replacements to coast redwoods.

## Sources of Funding

Our work was funded by the Santa Clara Valley Water District (CA, USA); and the Cities of Palo Alto (CA, USA) and Mountain View (CA, USA).

## Contributions by the Authors

L.L.N. and C.B. primarily collaborated in the drafting of the manuscript. L.L.N. primarily oversaw the analysis and interpretation of the data. C.B. and L.R.O. primarily collaborated in the design and acquisition of data. L.R.O. also provided critical revisions throughout the drafting of the manuscript.

## Conflict of Interest Statement

None declared.
